# The *Papaver* Self-Incompatibility Pollen *S*-Determinant, *PrpS*, Functions in *Arabidopsis thaliana*

**DOI:** 10.1016/j.cub.2011.12.006

**Published:** 2012-01-24

**Authors:** Barend H.J. de Graaf, Sabina Vatovec, Javier Andrés Juárez-Díaz, Lijun Chai, Kreepa Kooblall, Katie A. Wilkins, Huawen Zou, Thomas Forbes, F. Christopher H. Franklin, Vernonica E. Franklin-Tong

**Affiliations:** 1 School of Biosciences, University of Birmingham, Edgbaston, Birmingham B15 2TT, UK

## Abstract

Many angiosperms use specific interactions between pollen and pistil proteins as “self” recognition and/or rejection mechanisms to prevent self-fertilization. Self-incompatibility (SI) is encoded by a multiallelic *S* locus, comprising pollen and pistil *S*-determinants [1, 2]. In *Papaver rhoeas*, cognate pistil and pollen *S*-determinants, PrpS, a pollen-expressed transmembrane protein, and PrsS, a pistil-expressed secreted protein [3, 4], interact to trigger a Ca^2+^-dependent signaling network [5–10], resulting in inhibition of pollen tube growth, cytoskeletal alterations [11–13], and programmed cell death (PCD) [14, 15] in incompatible pollen. We introduced the *PrpS* gene into *Arabidopsis thaliana*, a self-compatible model plant. Exposing transgenic *A. thaliana* pollen to recombinant *Papaver* PrsS protein triggered remarkably similar responses to those observed in incompatible *Papaver* pollen: *S*-specific inhibition and hallmark features of *Papaver* SI [11–15]. Our findings demonstrate that *Papaver PrpS* is functional in a species with no SI system that diverged ∼140 million years ago [16]. This suggests that the *Papaver* SI system uses cellular targets that are, perhaps, common to all eudicots and that endogenous signaling components can be recruited to elicit a response that most likely never operated in this species. This will be of interest to biologists interested in the evolution of signaling networks in higher plants.

## Highlights

► PrpS, a *Papaver* SI determinant, functions in *Arabidopsis thaliana* pollen ► A “self” interaction with PrsS reveals *Papaver* SI hallmark features in *A. thaliana* ► The first evidence for transfamily functionality of an SI system (>140 my apart) ► Evidence of recruitment of signaling components for novel SI function

## Results and Discussion

### Expression of PrpS-GFP in *Arabidopsis thaliana* Pollen

**Figure 1:**
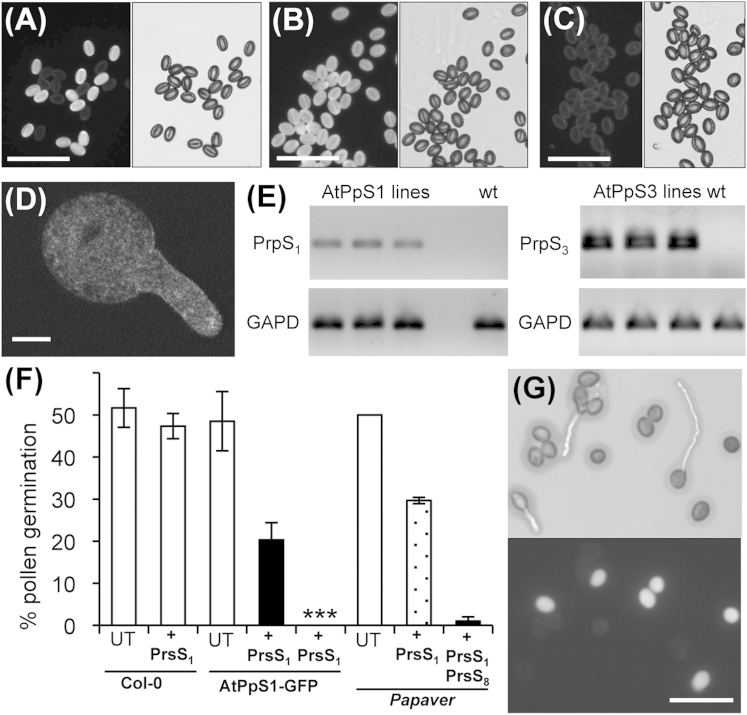
Expression of PrpS in Transgenic *Arabidopsis thaliana* (A) Fifty percent of pollen grains in *A. thaliana* lines *AtPpS1* hemizygous for PrpS_1_-GFP expression exhibit GFP fluorescence (left); brightfield image, right. (B) GFP fluorescence is observed in all pollen grains in homozygous *A. thaliana AtPpS1* line (left); brightfield image, right. (C) No GFP fluorescence is observed in *A. thaliana* wild-type pollen grains (left); brightfield image, right. (D) Confocal image of a PrpS_1_-GFP-expressing pollen tube. (E) RT-PCR to show expression of PrpS in *A. thaliana AtPpS1* and *AtPpS3* lines; WT, wild-type Col-0; GAPD was a loading control. (F) Quantification of inhibition of pollen germination of a hemizygous line of *AtPpS1* by PrsS_1_. Control pollen had high germination (white bars): untreated (UT), Col-0 pollen was unaffected by addition of PrsS_1_ (+PrsS_1_). Addition of PrsS_1_ to hemizygous GFP-expressing *AtPpS1* pollen (+PrsS_1_) had reduced pollen germination (black bar). When only GFP-expressing pollen were measured for this latter treatment (+PrsS_1_), no germination was observed (^∗∗∗^). *Papaver* pollen (from a plant haplotype *S_1_S_8_*): untreated (UT) had high germination, addition of PrsS_1_ inhibited half of the pollen, and addition of PrsS_1_ and PrsS_8_ gave inhibition of all pollen. (G) Pollen grains from a hemizygous *AtPpS1* line. Those not expressing PrpS_1_-GFP germinate and grow in the presence of PrsS_1_, whereas those exhibiting GFP fluorescence do not. Scale bars in (A), (B), (C), and (G) represent 100 μm; scale bar in (D) represents 10 μm. Error bars indicate ±SEM.

Transgenic lines from self-compatible *A. thaliana* ecotype Columbia (Col-0) were generated by introducing *PrpS_1_-GFP* (line *AtPpS1*) or *PrpS_3_-GFP* (line *AtPpS3*) under the control of the pollen-specific promoter *ntp303p* (see Supplemental Experimental Procedures available online). Transgenic lines in the T_2_ generation that segregated 3:1 were identified and pooled pollen assessed for GFP-expression. Two-thirds of the pollen was expected to be GFP-positive; 63.5% GFP expression was observed (n = 300). When pollen from individual plants was analyzed, pollen segregated either 50% or 100% for GFP-expression (n = 2,000, Figures 1A and 1B), consistent with them being hemizygous or homozygous for the insert; untransformed Col-0 pollen had low autofluorescence (Figure 1C). PrpS-GFP localized predominantly at the plasma membrane in pollen tubes (Figure 1D) as previously shown in *Papaver* pollen [4]. Expression of the *PrpS_1_/PrpS_3_* transgenes in these lines was confirmed using RT-PCR; transcripts were not detected in untransformed Col-0 plants (Figure 1E).

### Expression of PrpS-GFP Is Sufficient to Allow PrsS-Induced *S*-Specific Inhibition of *AtPpS* Pollen

To determine whether PrpS was functional in *A. thaliana*, we adapted the in vitro self-incompatibility (SI) bioassay system used for *Papaver* SI [3]. Transgenic pollen from lines *AtPpS1/AtPpS3* was grown in vitro and recombinant *Papaver* PrsS proteins added. If PrpS functions and utilizes a similar signaling network in *Arabidopsis*, this interaction should trigger *S*-specific pollen inhibition in pollen expressing PrpS-GFP. We tested whether this was the case (Figures 1F and 1G). Recombinant PrsS_1_ did not affect Col-0 pollen germination but reduced pollen germination from hemizygous *AtPpS1* pollen by 42% (n = 300). When only pollen expressing GFP was assessed after addition of PrsS_1_, none of these pollen grains germinated (Figures 1F and 1G, ^∗∗∗^p < 0.0001, n = 300). This correlation of GFP expression and pollen inhibition by PrsS_1_ demonstrates that PrsS_1_ inhibits *AtPpS1* pollen expressing PrpS_1_-GFP. This suggests that expression of PrpS_1_ in *Arabidopsis* pollen is sufficient to allow inhibition of pollen germination by PrsS_1_. Using *Papaver* pollen (from plants haplotype *S_1_S_8_)* confirmed that PrsS_1_ was functional (Figure 1F). Addition of PrsS_1_ partially reduced germination (p = 0.022, n = 300), addition of both PrsS_1_ and PrsS_8_ achieved complete inhibition (p = 0.009, n = 300).

**Figure 2:**
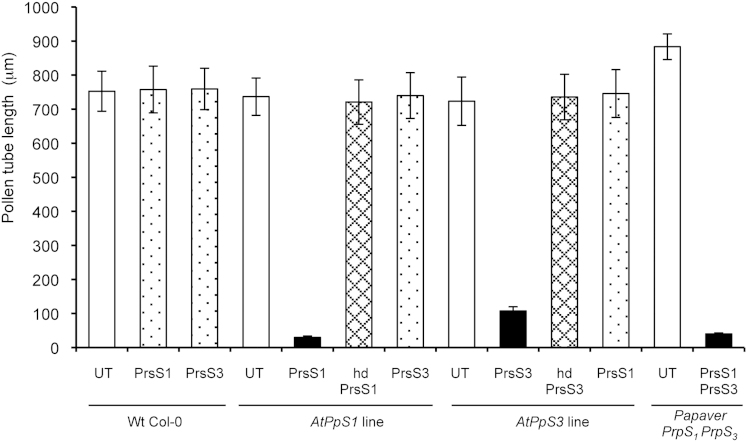
*S*-Specific Inhibition of Pollen Tube Growth in *A. thaliana* Pollen Expressing PrpS-GFP by Addition of Cognate PrsS Pollen tube lengths from homozygous lines *AtPpS1* and *AtPpS3* were measured after addition of PrsS_1_ and PrsS_3_. Untreated pollen tubes (UT, white bars) grew long; PrsS_1_ specifically inhibited pollen from line *AtPpS1* (black bar) and not pollen from *AtPpS3* or Col-0 (speckled bars); PrsS_3_ specifically inhibited pollen from line *AtPpS3* (black bar) and not pollen from *AtPpS1* or Col-0 (speckled bars). Heat-denatured PrsS (hd; cross-hatched bars) had no effect on pollen tube length. Untreated *Papaver* pollen from a plant haplotype *S_1_S_8_* (UT, white bar) had long pollen tubes, and addition of PrsS_1_ and PrsS_8_ gave strong inhibition (black bar). Error bars indicate ±SEM.

We next tested lines *AtPpS1* and *AtPpS3* homozygous for PrpS-GFP expression for *S*-specific inhibition of pollen tube growth by adding PrsS_1_ or PrsS_3_ (Figure 2). Col-0 pollen tube lengths were not significantly different from untreated transgenic lines after addition of PrsS_1_ or PrsS_3_ (p = 0.87, 0.89, n = 120). When PrsS_1_ was added to *AtPpS1* pollen, pollen tubes were significantly inhibited (>95% shorter compared to untreated controls, ^∗∗∗^p < 0.0001, n = 120). Similar results were obtained for PrsS_3_ addition to *AtPpS3* pollen (^∗∗∗^p < 0.0001; Figure 2). Inhibition of transgenic pollen was *S*-allele-specific, as when PrsS_3_ was added to *AtPpS1* pollen, no inhibition was observed compared to untreated controls (p = 0.95, n = 120); likewise, when PrsS_1_ was added to *AtPpS3* pollen, pollen tube lengths were not significantly different from untreated controls (p = 0.66, n = 120, Figure 2). Heat-denatured (biologically inactive) PrsS proteins had no effect. These data are consistent with the idea that PrpS expression in *A. thaliana* pollen is sufficient for an SI response (inhibition of “self” pollen) to be elicited. Control *Papaver* pollen from plants with haplotypes *S_1_S_3_* was inhibited (96% shorter than untreated, n = 120; ^∗∗∗^p < 0.0001) after addition of PrsS_1_ and PrsS_3_ (Figure 2).

### *A. thaliana* Pollen Expressing PrpS-GFP Exhibits *S*-Specific Actin Alterations after Addition of PrsS

**Figure 3:**
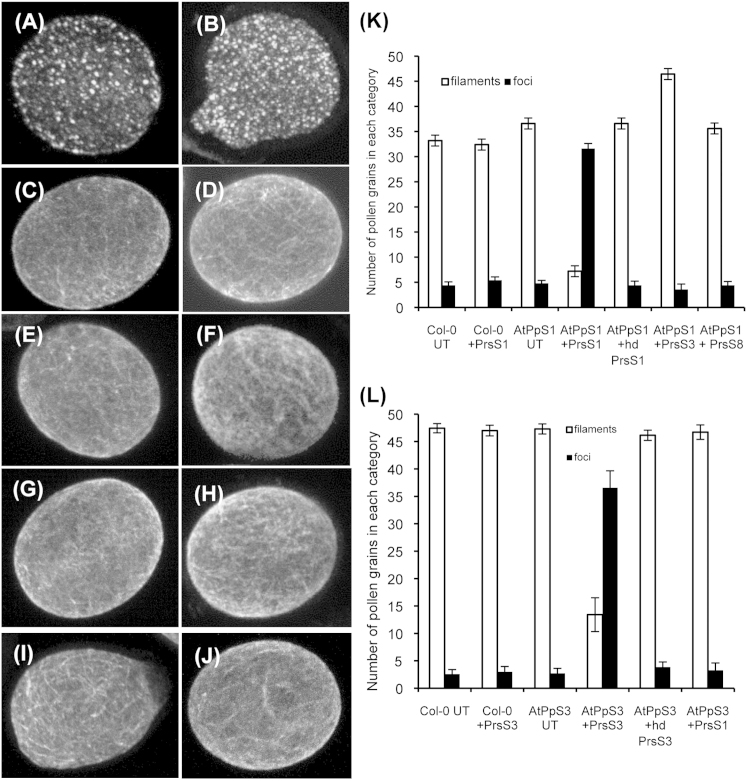
Actin Foci Are Stimulated in an *S*-Specific Manner in *A. thaliana AtPpS* Pollen by Cognate PrsS (A–J) F-actin was visualized using rhodamine-phalloidin and confocal imaging. (A and B) Typical punctate actin foci observed 3 hr after addition of PrsS_1_ to an *AtPpS1* pollen grain (A) and PrsS_3_ to an *AtPpS3* pollen grain (B). (C–F) Controls with normal actin arrays: untreated *AtPpS1* (C) and untreated *AtPpS3* pollen grains (D); “compatible” combinations (E and F), PrsS_8_ added to an *AtPpS1* pollen grain (E), PrsS_1_ added to an *AtPpS3* pollen grain (F), and heat-denatured PrsS_1_ and PrsS_3_ did not induce actin foci in *AtPpS1* and *AtPpS3* respectively (G and H). (I and J) Normal actin arrays were observed in wild-type Col-0 pollen grain untreated (I) or after addition of PrsS_1_ (J). (K and L) Quantitation of F-actin foci and normal filamentous actin arrays in pollen from the *A. thaliana AtPpS1* lines (K), pollen from the *A. thaliana AtPpS3* lines (L), and Col-0 acted as a control. White bars show normal actin filament arrays (as in C–F); black bars show punctate actin foci (as in A and B). Error bars indicate ±SEM.

We next investigated whether expression of PrpS in *A. thaliana* pollen was sufficient to induce similar intracellular responses to those elicited in incompatible *Papaver* pollen [7] by adding incompatible recombinant PrsS. A hallmark feature of *Papaver* SI is the *S*-specific formation of punctate actin foci [11, 12]. Punctate actin foci were formed when PrsS_1_ was added to *AtPpS1* pollen (Figure 3A); a similar response was observed in *AtPpS3* pollen after addition of PrsS_3_ (Figure 3B). Untreated pollen from these lines had normal filamentous actin organization (Figures 3C and 3D), and they retained this actin configuration after addition of compatible combinations of PrsS (*AtPpS1* with PrsS_8_, Figure 3E; *AtPpS3* with PrsS_1_, Figure 3F). When heat-denatured PrsS were used in an incompatible combination (Figures 3G and 3H), no actin foci were formed. Untransformed Col-0 pollen exhibited normal actin configuration (Figure 3I), and when PrsS_1_ was added to this pollen, no foci were formed (Figure 3J). This demonstrates that PrsS affects actin organization of *AtPpS1* and *AtPpS3* pollen specifically when used in a cognate allelic combination. Quantification (Figures 3K and 3L) showed that filamentous actin is the predominant phenotype, except for the combination of cognate recombinant PrsS with PrpS pollen (*AtPpS1* pollen with PrsS_1_ added, and *AtPpS3* pollen with PrsS_3_). These two samples were significantly different from untreated pollen (^∗∗∗^p < 0.0001, n = 250; ^∗∗∗^p < 0.0001, n = 350). All other comparisons were not significantly different from untreated controls or Col-0, for example, *AtPpS1* pollen with PrsS_8_ added, compared to untreated pollen (p = 0.85, n = 250). Thus, formation of punctate actin foci is induced in an *S*-allele-specific manner in *Arabidopsis PrpS*-expressing pollen by *Papaver* PrsS. As expression of PrpS in *A. thaliana* pollen is sufficient to elicit this key hallmark feature of *Papaver* SI, it suggests that all the signaling components necessary for this “*Papaver*-like” SI response are present.

### *S*-Specific Death Is Induced by PrsS in *A. thaliana* pollen Expressing PrpS-GFP

**Figure 4:**
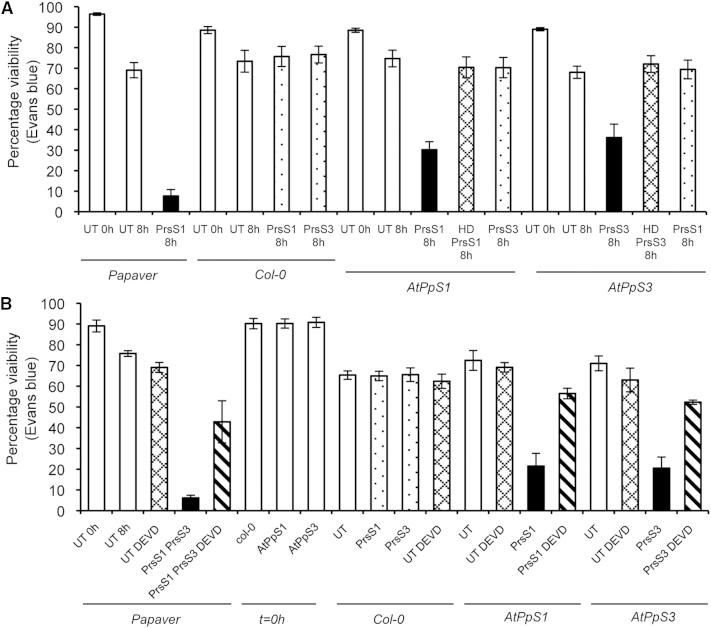
Death Involving a DEVDase/caspase-3-like Activity Is Stimulated in an *S*-Specific Manner in *A. thaliana* Expressing PrpS_1_-GFP or PrpS_3_-GFP (A) Quantitation of Evans blue staining 8 hr after addition of PrsS (percent viability). All untreated (UT, white bars) pollen at time 0 had high viability; this was slightly reduced after 8 hr. Addition of PrsS_1_ and PrsS_3_ to *Papaver* pollen carrying PrpS_1_ and PrpS_3_ resulted in low viability (black bar); addition of PrsS (speckled bars) to *A. thaliana* Col-0 pollen did not affect viability; addition of PrsS_1_ to *AtPpS1* pollen and PrsS_3_ to *AtPpS3* pollen reduced viability (black bars). Heat-denatured PrsS (HD-PrsS, cross-hatched bars) did not affect viability. (B) Pretreatment with Ac-DEVD-CHO prevents *S*-specific death of *A. thaliana* pollen. Quantitation of percent viability (Evans blue) after pretreatment with Ac-DEVD-CHO and addition of PrsS. Untreated (UT, white bars) pollen had high viability. Addition of the caspase-3 inhibitor, Ac-DEVD-CHO to UT pollen (UT DEVD, cross-hatched) had no effect. Addition of PrsS_1_ and PrsS_3_ to *Papaver* pollen carrying PrpS_1_ and PrpS_3_ resulted in low viability (black), and pretreatment with Ac-DEVD-CHO prior to addition of PrsS_1_ or PrsS_3_ (diagonal bars) resulted in higher viability. Addition of PrsS to *A. thaliana* Col-0 pollen did not affect viability (stippled bars); addition of PrsS_1_ to *AtPpS1* pollen and PrsS_3_ to *AtPpS3* pollen reduced viability (black bars). Pretreatment with Ac-DEVD-CHO prior to addition of PrsS_1_ and PrsS_3_ (diagonal bars) resulted in higher viability. Error bars indicate ±SEM.

A key feature of SI in *Papaver rhoeas* is the triggering of programmed cell death (PCD) in incompatible pollen [14, 15]. To provide further evidence for PrpS elicitation of a *Papaver*-like SI response, we investigated whether death was triggered in *AtPpS1* and *AtPpS3* pollen after addition of PrsS, by assessing viability of pollen using Evans blue at 8 hr (Figure 4A). PrsS_1_ and PrsS_3_ activity was demonstrated by addition to *Papaver* pollen from plants haplotype *S_1_S_3_*; this gave an 89% loss of viability compared to untreated pollen (^∗∗∗^p < 0.0001, n = 300, Figure 4A). Untransformed Col-0 pollen viability was not significantly affected after addition of PrsS_1_ or PrsS_3_ (p = 0.71, p = 0.60, n = 500). Addition of PrsS_1_ to *AtPpS1* pollen resulted in a 60% reduction in pollen viability compared to untreated controls (^∗∗∗^p < 0.0001, n = 500). Similar results were obtained with PrsS_3_ added to *AtPpS3* pollen (p < 0.0001, n = 500). Loss of viability was *S*-allele-specific; when PrsS_3_ was added to *AtPpS1* pollen, and when PrsS_1_ was added to *AtPpS3* pollen, there was no significant difference in viability compared to untreated pollen (p = 0.48, 0.83 respectively, n = 500). As expected, heat-denatured PrsS had no effect. Thus, PrsS can trigger *S*-specific death in *A. thaliana* pollen expressing PrpS-GFP, specifically in combination with cognate (“self”) PrsS.

### *S*-Specific Death Induced by PrsS Involves a DEVDase/caspase-3-like Activity

Although Evans blue demonstrates cell death, it does not indicate whether PCD is involved. As *Papaver* SI relies on a DEVDase/caspase-3-like activity [14, 15], we assessed whether a similar activity was involved in the death of *PrpS*-expressing *A. thaliana* pollen, by adding Ac-DEVD-CHO, a caspase-3 inhibitor before addition of PrsS (Figure 4B). PrsS_1_ and PrsS_3_ added to *Papaver* pollen carrying *PrpS_1_* and *PrpS_3_* resulted in 91% loss in viability compared to untreated pollen (^∗∗∗^p < 0.0001, n = 300); pretreatment with Ac-DEVD-CHO resulted in significantly higher viability at 8 hr (p < 0.0001, n = 300). Ac-DEVD-CHO had no effect on *Arabidopsis* pollen viability (p = 0.66 for Col-0, p = 0.60 for *AtPpS1*, 0.23 for *AtPpS3*). Pretreatment of pollen with Ac-DEVD-CHO before PrsS addition resulted in significantly higher viability compared to samples with PrsS_1_ or PrsS_3_ added alone. *AtPpS1* pollen viability was not significantly different to that in the presence of Ac-DEVD-CHO alone (p = 0.065, NS, n = 300); for *AtPpS3* homozygotes, viability was only 17% less than pollen from the same line in the presence of Ac-DEVD-CHO alone (p = 0.13, NS, n = 300). Prevention of PrsS-induced death of *AtPpS1* and *AtPpS3* pollen by Ac-DEVD-CHO provides strong evidence that PrpS triggers a functional “*Papaver*-like” SI response involving a DEVDase/caspase-3-like activity in *A. thaliana* pollen. It also suggests that similar signaling networks to those used in the *Papaver* SI response [14, 15, 17] are used in *AtPpS* pollen that result in pollen PCD.

Together, our findings demonstrate that although the SI determinants in *Papaver* are completely distinct from those identified at a molecular level in other SI systems, PrpS functions as an *S*-determinant when transferred into a self-compatible species from a distantly related genus. *Papaver* belongs to the most basal order in the eudicots, the Ranunculales, whereas *Arabidopsis* belongs to the Brassicales, with ∼140 million years evolutionary distance between them [16]; see Figure S1. So far the only functional transfer of *S*-determinants has been between closely related species. Interspecific and intergeneric transfer of orthologs of *Brassica S*-determinants [18–20] from self-incompatible *A. lyrata* and *Capsella grandiflora* [21, 22] into self-compatible *A. thaliana* is sufficient to confer SI [23, 24]. This provided good evidence that *A. thaliana* has all the components required for a *Brassica*-type SI to be elicited, though the detailed mechanisms are not yet fully elucidated. Although these are important demonstrations, *A. thaliana* and *A. lyrata* diverged only ∼5 million years ago (mya) [21], *Arabidopsis* and *Capsella* separated ∼6.2–9.8 mya [25], and self-compatibility originated very recently (<0.5 mya [26]). Thus, despite the importance of these studies, major insights into the evolution of SI signaling across angiosperm families is lacking as a result of their close relationship and their possession of a mechanistically common SI system. *P. rhoeas* has a gametophytic SI system that is genetically controlled in a completely different manner from the sporophytic SI system in the Brassicaceae. These two SI systems are thought to have evolved completely independently [27], and there is no evidence of a shared ancestral SI system, because *A. thaliana* does not possess orthologs of the *Papaver S*-determinants. Here we show that, despite the huge evolutionary distance and lack of a common SI system, transgenic *A. thaliana* pollen expressing *PrpS-GFP* is not only rejected but also displays remarkably similar cellular responses to that triggered in incompatible *Papaver* pollen.

Our data provide good evidence that *A. thaliana* recruits existing proteins to form new signaling networks to trigger a function (SI) that does not normally operate in this species. As a *Papaver*-like SI response, involving formation of punctate actin foci and PCD involving a caspase-3-like/DEVDase activity has not been observed in the *Brassica*-type SI response, it suggests that the PrpS-PrsS interaction is sufficient to specify a particular downstream signaling network to obtain this outcome. Studies on the evolution of self-/non-self-recognition systems has largely focused on the receptors and ligands involved in recognition [28, 29] rather than the signaling networks triggered by their interaction. Our findings suggest either conservation of a signaling system or recruitment of core signaling components to mediate downstream SI responses and will open up debate about how these systems evolved. It appears that the *Papaver* SI system works in *A. thaliana* due to “multitasking” of endogenous components that can “plug and play” to act in signaling networks that they do not normally operate in, to provide a specific, predictable physiological outcome. This has previously been shown in other systems (see [30–32]), and a compelling argument has been made for the utilization of convergent evolution in innate immune pathways [33]. Our findings confirm postulated parallels between SI and plant-pathogen resistance [29, 34] and the idea that SI may utilize these signaling networks. Our data suggest that the signaling networks and cellular targets for *Papaver* SI are “universal,” unspecialized, and ancient and may be present in a wide range of angiosperm species. We suggest that this is a likely explanation of why PrpS functions in *A. thaliana* pollen.

### Conclusions

Expression of the *Papaver* male *S*-determinant, PrpS, in *A. thaliana* pollen is sufficient to allow it to differentiate between different allelic products of the *Papaver* female *S*-locus determinant, PrsS, and trigger an *S*-allele specific rejection response when it encounters cognate PrsS protein. Functionality in a highly diverged compatible species has implications for our perspective of evolution of signaling networks in higher plants. Moreover, wide transgenera functionality of the *Papaver* SI system opens up the possibility that, assuming that PrsS can also be functionally expressed, transferral of these *S*-determinants may, in the longer-term, provide a tractable SI system to transfer to crop plants. This has implications for solving food security issues, by allowing breeding of superior F1 hybrid plants more easily and cheaply.

## Supplemental Information

Document S1. Figures S1 and S2, Table S1, and Supplemental Experimental Procedures
