# Supplementary material for: The Papaver Self-Incompatibility Pollen S-Determinant, PrpS, Functions in Arabidopsis thaliana
Source: Curr Biol. 2012 Jan 24;22(2):154–9. doi: 10.1016/j.cub.2011.12.006 (PMC3695568; doi:10.1016/j.cub.2011.12.006)
Supplement: Document S1. Figures S1 and S2, Table S1, and Supplemental Experimental Procedures [file mmc1.pdf]

Current Biology, Volume *22*

Supplemental Information

The *Papaver* Self-Incompatibility Pollen *S*-Determinant, *PrpS*, Functions in *Arabidopsis thaliana*

Barend H.J. de Graaf, Sabina Vatovec, Javier Andrés Juárez-Díaz, Lijun Chai, Kreepa Kooblall, Katie A. Wilkins, Huawen Zou, Thomas Forbes, F. Christopher H. Franklin, and Vernonica E. Franklin-Tong

Supplemental Inventory

1. Supplemental Figures and Tables

Figure S1, related to Discussion

Figure S2, related to Supplemental Experimental Procedures

Table S1, related to Supplemental Experimental Procedures

2. Supplemental Experimental Procedures

3. Supplemental References

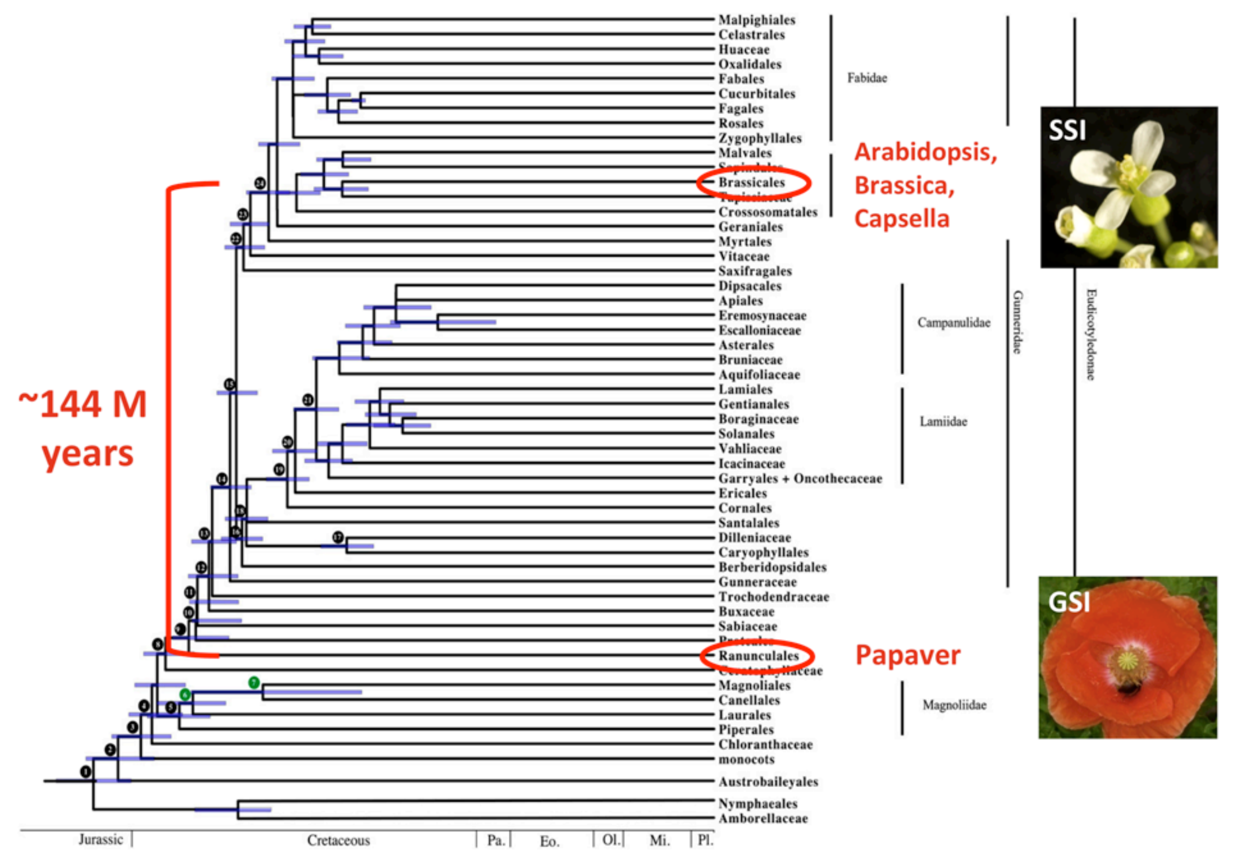

Phylogeny taken from: Bell,Soltis & Soltis (2010). The age and diversification of the angiosperms re-revisited. *American Journal of Botany* 97, 1296-1303.

Figure S1. The *Papaver* Pollen *S*-determinant, *PrpS*, Can Allow a Self-Rejection Response in Pollen of a Self-Compatible Species that Diverged ~144 Million Years Ago

A. thaliana (top right hand image) is self-compatible with a different ancestral self-incompatibility (SI) system to *Papaver* (bottom right hand image), which uses Gametophytic SI, GSI). *A. thaliana* diverged from *A. lyrata* (which uses a Sporophytic SI (SSI) system with orthologs of the *Brassica S*-determinants) ~ 5 million years ago. *Papaver* is a basal eudicot, placed in the Ranunculales, while *Arabidopsis* belongs to the Brassicales; recent phylogenetic analysis estimates that there is ~144 million years evolutionary distance between them. Despite this large evolutionary distance and difference in SI systems, here we show that downstream signaling components and targets can be recruited in *A. thaliana* pollen expressing PrpS to give a "*Papaver-like*" SI response upon interaction with PrsS, the pistil determinant.

Phylogeny taken from: Bell,Soltis & Soltis, The age and diversification of the angiosperms re-revisited. *American Journal of Botany* 97, 1296 (2010). © The Botanical Society of America.

Figure S2. Cartoon Representation of the Chimeric Transgenes Cassettes Cloned into pGreen 0029 Binary Vectors that Were Used for Transformation of *Arabidopsis thaliana,* Driving PrpS-GFP Protein Fusion Specifically in Pollen

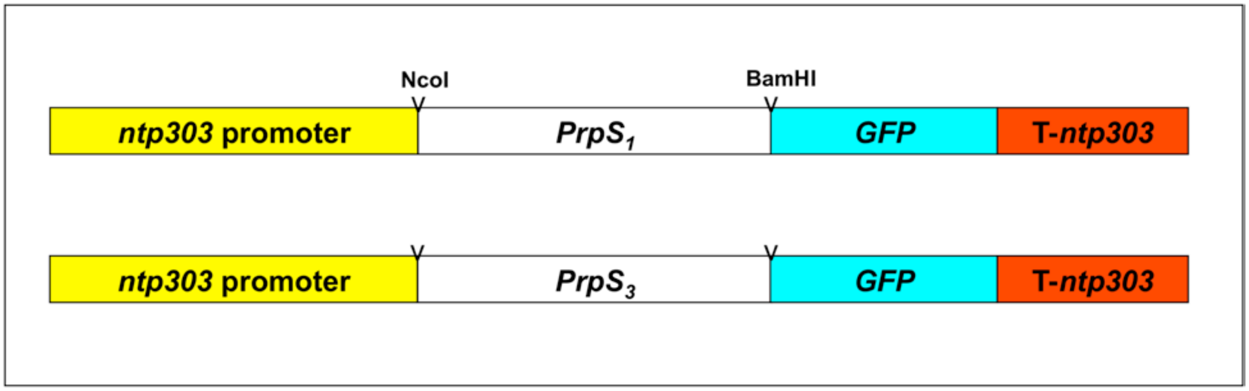

Table S1. Oligonucleotides Used for T-DNA Gene Construction and Expression Analysis

**T-DNA gene construction**		
PpS_1;	PrpS1-5-NcoI	5’-T ACC ATG GCC CGA AGT GGA AGT GTT G-3’
	PrpS1-3-BHI	5’- AT GGA TCC AGC TTG AGT TAT AAG ATG AGG GG -3’
PpS_3;	PrpS3-5-NcoI	5’-TC CCC ATG GCA CGA AAT AGA CAT GC-3’
	PrpS3-3-BHI	5’-TAT GGA TCC AGC CTC ATT AGG ACA TGG-3’
**Expression analysis**		
PpS_1;	PpS1-FW	5’-CC ATG CCC CGA AGT GGA AGT GTT G-3’
	PpS1-REV	5’-CC TTA AGC TTG AGT TAT AAG ATG AGG GGA ATC C-3’
PpS_3;	PpS3-FW	5’-CC ATG CTC TTA CGT GGA AAG ACC-3’
	PpS3-REV	5’-GG CTG CAG AAG TGG CTT CAT C-3’
GAPD;	GAPD FW	5’-CAC TGA CAA AGA CAA GGC TGC AGC-3’
	GAPD REV	5’-CCT GTT GTC GCC AAC GAA GTC AG-3’

Supplemental Experimental Procedures

T-DNA Construction, Plant Transformation, Screening, and Selection

The coding regions of the *Papaver rhoeas* pollen *S*-determinant *$PrpS_1$* and *$PrpS_3$* (Wheeler et al. 2009) were amplified by PCR (**Supplemental Table 1**; see below), without stop codons, after which the C-termini were fused in frame to the coding sequence of Green Fluorescent Protein (GFP). The *ntp303* pollen promoter and *ntp303* terminator sequence (T-*ntp303*; (*1, 2*) were used for pollen-specific expression of both fusion proteins (see **Supplemental Figure 2**). Both chimeric genes were subcloned into pGreenI 0029 binary vectors (John Innes Centre, U.K.). The T-DNA of this vector carries the *ntpII* kanamycin resistance gene, which is used for selection of transgenic plants after transformation. Transformation of *Arabidopsis thaliana* (ecotype Columbia, Col-0) was carried out by standard 'dipping' procedures (*3*). T_0 seeds were collected and screened for transformants (T_1) on kanamycin (kan)-selective MS_{30} plates (30 $mg.ml^{-1}$ kanamycin). Pollen from independent kan-resistant T_1 *A. thaliana* plants was screened for single T-DNA insertions by checking for GFP expression in half of the pollen population. Pollen was also scored for 'low', 'medium' and 'high' PrpS-GFP expressor plant lines.

After selfing of the primary T_1 *A. thaliana* plant lines expressing the *Papaver* pollen *S*-determinants *$PrpS_1$* or *$PrpS_3$* (so named *AtPpS1* and *AtPpS3* respectively), the segregation of transgenes in these lines was analysed by analysing the seeds on MS kan_{30} plates. Transgenic lines were assessed for kan selection by counting the numbers of seedlings that had grown in the presence of kanamycin (n=100 for at least three independent sets). Most transgenic plant lines, independent of their GFP protein expression levels in pollen, showed the expected 3:1 (kan resistant: kan sensitive) Mendelian segregation. Kan-resistant plants were transferred to soil and grown to flowering. Pollen from individual plants and from several generations (T_2-T_5) was checked for heterozygosity and homozygosity for PpS_1-GFP or PpS_3-GFP transgene expression before using for experiments. For experimental analysis, pollen was collected and pooled from several plants.

Assessment of GFP-Expressing Pollen

Pollen from flowering Kan-resistant plants was assessed for GFP expression. Randomised samples of 100 pollen grains or pollen tubes in a field of view (x10 objective) were analysed using a Nikon Tε400 microscope. Pollen grains were either counted using both bright field and fluorescence, using a narrow band-pass filter for FITC (492/18x Single Band Blue exciter) to avoid autofluorescence from pollen grains. Wild-type Col-0 pollen exhibited virtually no autofluorescence using the same settings as used for GFP lines. Imaging was done using a Nikon DS-Qi1MC monochrome cooled CCD camera and Nikon Imaging Software (NIS) elements BR3.2 program using a standardized exposure time and gain for all assessments.

Arabidopsis thaliana Pollen Tube Culture and the "In Vitro SI Bioassay" Adapted for Use in *Arabidopsis* as an Assay for *S*-determinant Function

Pollen from open flowers (stage 13; (*4*)) was cultured *in vitro* for experimental analysis. Two liquid pollen germination medium (PGM) recipes were used: (1) PGM comprising: 5 mM $CaCl_2.2H_2O$, 0.01% (w/v) H_3BO_3, 5 mM KCl, 1 mM $MgSO_4$ $7H_2O$, and 10% (w/v) Sucrose, pH 7.5; ref (*5*), or (2) PMG comprising: 0.01% (w/v) H_3BO_3, 1 mM $CaCl_2.2H_2O$, 1 mM $Ca(NO_3)_2$, 1 mM $MgSO_4$, and 18% sucrose, pH 7.0; ref (*6*). Pollen was released from fresh open flowers by vortexing in PGM and resuspension of the pollen pellet in PGM or by dipping flowers into PGM.

We have used *in vitro* bioassays for demonstration of PrpS function to complement the strategy of stable transformations. These bioassays have been routinely used to demonstrate *S*-specific function in *Papaver* and full details are to be found in the Supplemental Methods of (*7*). For SI treatments, recombinant PrsS proteins were produced by cloning the nucleotide sequences specifying the mature peptide of the S_1 and S_3, alleles of PrsS, the pistil *S*-determinant (pPRS100, pPRS300) into the expression vector pMS119 as described previously in (*8*). Expression and purification of the proteins was performed as described previously (*9*). Pollen from mature non-transgenic Col-0 and transgenic *A. thaliana* flowers was collected into liquid PMG. Pollen was usually pre-germinated for ~45 min before application of the treatments (see below). For controls, pollen of *Papaver rhoeas* was grown in parallel in liquid *Papaver* PGM (0.01% H_3BO_3, 0.01% KNO_3, 0.01% $Mg(NO_3)_2$-$6H_2O$, 0.036% $CaCl_2x2H_2O$ and

13.5% sucrose) at 23°C. For the SI *in vitro* bioassays carried out here, we used recombinant PrsS proteins (final working concentration 10-30 µg.ml^{-1}) to provide an incompatible combination with the transgenic *A. thaliana* pollen expressing PpS_1-GFP or PpS_3-GFP. Non-cognate allelic combinations, addition of germination medium and of heat-denatured recombinant $PrsS_1$ proteins, which are biologically inactive, acted as negative controls. Additional positive controls comprising *Papaver* pollen with appropriate combinations of recombinant PrsS were used in parallel for each experiment to verify the activity and specificity of the proteins. Pollen was cultured in the presence of PrsS proteins for 2 h to 16 h for inhibition assays, for 3 h for assays assessing formation of punctate actin foci, and for 8 h for viability assays. Statistical tests comprised two-way comparisons between pairs of data using a student's t-test or X^2 analysis

Pollen Tube Length Measurements

Pollen tubes were grown and treated as described above, and incubated at 23°C for 2 h or 16 h, fixed in 4% paraformaldehyde and mounted in Tris-buffered saline (pH 7.6). Pollen tubes were imaged and pollen tube lengths measured using a Nikon eclipse Tε300 inverted microscope, Nikon DS-Qi1MC monochrome cooled CCD camera, and Nikon Imaging Software (NIS) elements BR3.2 program. Either 20 or 100 pollen tubes were measured for each treatment and the mean length determined from a total of 3-7 independent experiments. Statistical analysis comprised of t-test analyses.

F-actin Imaging and Quantification

Pollen grains were fixed in 400 µM 3-maleimodobenzoic acid N-hydroxysuccinimide ester (MBS, Pierce) for 6 min, followed by 2% formaldehyde (1.5 h, 4°C). Pollen tubes were washed in TBS (50 mM Tris pH 7.6, 200 mM NaCl) then permeabilized with TBS + 0.1% Triton X-100 for 40 min. F-actin was labeled by addition of 66 nM rhodamine-phalloidin (Invitrogen); pollen tubes mounted on slides with 5 µL of Vectashield (Vector Laboratories, USA) and imaged using fluorescence microscopy;see (*10*). F-actin imaging and quantitation was performed using epifluorescence with a Nikon Tε300 and a standard Texas Red filter (Chroma Technology Corp.). Pollen tubes were scored as either "normal" F-actin configuration, "actin foci", which are a typical marker induced by SI, and "intermediate" (not in either category). 50 pollen tubes for each of 5 replicates were scored (i.e. a total of 250 pollen grains scored for each treatment). Images were captured using Confocal laser scanning microscopy using a Leica SP2 microscope with a 63x oil immersion objective with the 543 nm laser (emission 565-600 nm). Full z-series sections of the pollen tubes were taken, with 0.5 µm thick optical sections. Images were saved as tiff files.

Viability Assays

A.thaliana *PpS_1-GFP* and *PpS_3-GFP* pollen was pre-germinated and recombinant $PrsS_1$/$PrsS_3$ added to samples; controls included untreated pollen as well as non-transgenic Col-0 pollen; *Papaver* pollen was used a a positive control. Pollen was left at 23°C for 8 h, after which 0.05 % Evans Blue was added for 10 minutes; controls at time 0 were also measured to assess any change in viability over the 8 h time period. After washing to remove excess dye, samples were mounted on microscope slides and pollen assessed for staining using brightfield microscopy (Nikon Eclipse Tε300). Counts were made of unstained (live) and dark-stained (dead) pollen grains and tubes. For each sample 100 pollen grains or pollen tubes were counted (5 replicates for *A.thaliana* PpS-GFP pollen, 3 replicates for *Papaver* pollen). Statistical analyses were performed using a X^2 test.

To test if a DEVDase was involved in mediating viability, pollen was germinated as described and pretreated by addition of 100 µM Ac-DEVD-CHO (Calbiochem) for 1 h prior to SI induction. Controls included untreated pollen and pollen only incubated with Ac-DEVD-CHO and pollen with SI induced without Ac-DEVD-CHO. After SI induction, pollen was left at 23°C for 8 h, after which 0.05 % Evans Blue was added for 10 minutes. After washing to remove excess dye, aliquots of resuspended pollen were mounted on microscope slides and pollen was visualised using brightfield microscopy (Nikon Eclipse Tε300). Counts were made of unstained (live) and dark-stained (dead) pollen grains and tubes (sample size 145, with at least 3 replicates). Statistical analysis was performed using a X^2 test.

PpS mRNA Expression Analysis

$PrpS_1$ and *$PrpS_3$* expression in *A. thaliana* non-transgenic Col-0 and transgenic *A. thaliana AtPpS* pollen was analysed by RT-PCR on cDNA prepared from mature flowers (*1*). Total RNA was extracted using RNAeasy Mini Kit (QIAGEN). Total RNA from flowers at anthesis was isolated after grinding in liquid nitrogen and processing as described by the manufacturer. After DNAse treatment (Invitrogen), isolated total RNA was used for cDNA synthesis (One-step RT-PCR kit, QIAGEN) and subsequently PCR using *Papaver $PrpS_1$* and *$PrpS_3$* gene specific primers (see **Supplemental Table 1**). Glyceraldehyde-3-phosphate dehydrogenase (GAPD) primers were used as a positive control. Oligonucleotides were obtained from Eurofins MWG Operon (Ebersberg, Germany).

Supplemental References

1. K. Weterings, J. Schrauwen, G. Wullems, D. Twell, *Plant Journal* 8, 55 (1995).
2. B. H. J. de Graaf *et al.*, *Nature* 444, 490 (2006).
3. S. J. Clough, A. F. Bent, *Plant Journal* 16, 735 (1998).
4. D. R. Smyth, Bowman, J.L. Meyerowitz, E.M. , *Plant Cell* 2, 755 (1990).
5. L. C. Boavida, S. McCormick, *Plant Journal* 52, 570 (2007).
6. H. Li, Y. Lin, R. M. Heath, M. X. Zhu, Z. Yang, *Plant Cell* 11, 1731 (1999).
7. M. J. Wheeler *et al.*, *Nature* 459, 992 (2009).
8. H. C. C. Foote *et al.*, *Proceedings of the National Academy of Sciences of the United States of America* 91, 2265 (1994).
9. K. Kakeda *et al.*, *Plant Cell* 10, 1723 (1998).
10. N. S. Poulter, C. J. Staiger, J. Z. Rappoport, V. E. Franklin-Tong, *Plant Physiology* 152, 1274 (2010).
